# Digital Elder Abuse Intervention for Early Detection of Abuse in Older Adults Living with Dementia

**DOI:** 10.1002/alz.092332

**Published:** 2025-01-09

**Authors:** Fuad Abujarad, Chelsea Edwards, Judith A. Neugroschl, Ula Hwang, Richard Marottoli

**Affiliations:** ^1^ Yale University, New Haven, CT USA; ^2^ Icahn School of Medicine at Mount Sinai, New York, NY USA; ^3^ Yale School of Medicine, New Haven, CT USA

## Abstract

**Background:**

Elder abuse (EA) is a major public health problem and older people living with dementia (PLWD) are not likely to self‐report EA. As a result, identification of EA remains low, and providers often miss the opportunity to identify EA during Emergency Department (ED) visits. We present a pilot study on adapting an evidence‐informed intervention to motivate PLWD to self‐report abuse despite existing cognitive challenges.

**Method:**

Our tablet‐based digital health intervention, VOICES, was developed to increase self‐reporting of EA among PLWD. VOICES uses educational content, screening, multimedia elements, and brief psychoeducational interviewing with a digital coach to motivate PLWD to report abuse on their own. VOICES’ success was previously demonstrated with at least 1,000 cognitively intact participants (ages 60+) in the ED and primary care setting and piloted with PLWD at a geriatric memory clinic (N = 30). This study evaluated using VOICES among PLWD (ages 60+ years) in the ED setting (N = 101). Participants were recruited, consented, and enrolled at Yale New Haven Health System in New Haven, Connecticut, USA. We used the Montreal Cognitive Assessment (MoCA) to assess cognitive ability of participants, excluding those with severe cognitive impairments.

**Result:**

Ninety‐nine older adults with MoCA scores between 14 and 25 used VOICES independently and completed post‐survey questions. Satisfaction was high across all participants. Of all participants, eight self‐reported EA. 75% of participants who self‐reported (6/8) were offered additional services following a social worker evaluation, and 12.5% of participants who self‐reported (1/8) were reported to Adult Protective Services for EA.

**Conclusion:**

Our findings suggest that not only is VOICES feasible (i.e., acceptable, practical, and satisfactory) for detecting EA in PLWD (MoCA scores 14‐25), but can provide additional services to those who would have otherwise gone discharged without intervention. VOICES may be able to be used for early detection and prevention of severe abuse for this high‐risk population. More research is needed to determine efficacy and long‐term outcomes of the benefits and harms.

